# Circadian PER1 controls daily fat absorption with the regulation of PER1-PKA on phosphorylation of bile acid synthetase

**DOI:** 10.1016/j.jlr.2023.100390

**Published:** 2023-05-18

**Authors:** Wenhao Ge, Qi Sun, Yunxia Yang, Zhao Ding, Junhao Liu, Jianfa Zhang

**Affiliations:** 1 Center for Molecular Metabolism, Nanjing University of Science & Technology, Nanjing, China; 2 Key Laboratory of Cardiovascular and Cerebrovascular Diseases, Bengbu Medical College, Bengbu, China

**Keywords:** *Per1*, bile acid and salts/biosynthesis, cholesterol 7-alpha hydroxylase, dietary fat, protein kinases, bile acid and salts

## Abstract

Several epidemiological studies suggest a correlation between eating time and obesity. Night eating syndrome characterized by a time-delayed eating pattern is positively associated with obesity in humans as well as in experimental animals. Here, we show that oil intake at night significantly makes more fat than that at day in wild-type mice, and circadian *Period 1* (*Per1*) contributes to this day–night difference. *Per1*-knockout mice are protected from high-fat diet–induced obesity, which is accompanied by a reduction in the size of the bile acid pool, and the oral administration of bile acids restores fat absorption and accumulation. We identify that PER1 directly binds to the major hepatic enzymes involved in bile acid synthesis such as cholesterol 7alpha-hydroxylase and sterol 12alpha-hydroxylase. A biosynthesis rhythm of bile acids is accompanied by the activity and instability of bile acid synthases with PER1/PKA-mediated phosphorylation pathways. Both fasting and high fat stress enhance *Per1* expression, increasing the fat absorption and accumulation. Our findings reveal that *Per1* is an energy regulator and controls daily fat absorption and accumulation. Circadian *Per1* controls daily fat absorption and accumulation, suggesting *Per1* is a potential candidate of a key regulator in stress response and the relevant obesity risk.

The relatively fixed feeding behaviors in all mammals, especially feeding time, seem to be adapted to their respective environment for each species’ survival as a result of evolution. Biological rhythms have been implicated in this adaptation since circadian clock controls multiple feeding-related systems, which display daily variations of locomotion activity, gastrointestinal motility, and activity of digestive enzymes (1, 2). Organs and tissues involved in food intake present a large number of genes that encode for important regulators of carbohydrates, lipids, and proteins metabolism and show oscillations in their expressions (3). Thus, these clock-controlled genes expression and feeding time are to allow the animal to predict food availability.

It is found that clock mutant mice have a greatly attenuated diurnal feeding rhythm (4), and the mice lost *Period1 (Per1*) and *Period2* (*Per2*) demonstrate completely arrhythmic locomotion and feeding time over 24 h (5). Inversely, the changes in timing of food and types of nutrient intake may lead to an uncoupling of peripheral oscillators from the central pacemaker (6, 7). Followed restricted feeding schedules, several physiological functions and variables occur before eating, preparing to anticipate food intake and being synchronized (8). Therefore, disrupted feeding time can induce a disruption of the circadian system which might produce unhealthy consequences. Several epidemiological studies suggest a correlation between feeding time and obesity both in human and experimental animals (9, 10), and total energy intake, dietary composition, and estimated energy expenditure cannot explain this association (11). The mechanism linking feeding time and body weight gain remains unclear.

Here, we show a novel mechanism of feeding time regulating body weight by tracing the intestinal absorption of fat in mice. We found that night eating significantly increased intestinal fat absorption through increasing the emulsification of lipids in wild-type (WT) mice, and this phenomenon was abolished in *Per1*-deficient mice. We identified that rhythmic *Per1* contributed to changing the size and composition of bile acid (BA) pool, and this occurred through a mechanism involving binding-mediated cholesterol 7alpha-hydroxylase (CYP7A1) and sterol 12α-hydroxylase (CYP8B1) activity and instability. We demonstrated that circadian *Per1* was an energy regulator and modulated intestinal absorption of fat. Our findings provide theoretical evidences for healthy dietary arrangements and raise novel therapeutic possibilities for human obesity.

## Materials and methods

### Animal studies

*Per1*^*−/−*^ and *Per2*^*−/−*^ mice were obtained from Dr CC Lee at Baylor College of Medicine, Houston, TX (12, 13). *Per1*^*−/−*^ and *Per2*^*−/−*^ mice on the 129SvEv background were bred onto the C57BL/6J (Jackson Laboratory, Bar Harbor, ME) background for 8–10 generations (N8–N10) according to standard genetic protocols. Mice were housed in a standard animal maintenance facility under a 12-h/12-h light/dark cycle (light on at 7 a.m., ZT0; lights off at 7 p.m., ZT12) and provided with food and water ad libitum. For circadian/diurnal studies, mice aged between 6 and 8 weeks were sacrificed every 4 h for 24 h. For diet-induced obesity studies, mice were fed a normal diet (ND) or high-fat diet (HFD) (catalog no.D12492, Research Diets, New Brunswick, NJ) starting at 4 weeks of age for 8 weeks. Food and water intake were measured for each cage (3–4 mice per cage) every 2 days at 9:00 a.m. Food and water intake were expressed as grams per day per body weight. All procedures were approved by the Animal Care and Use Committee at Nanjing University of Science and Technology (NJUST-AC-05-1217).

### Bomb calorimetry

For the estimation of the amount of energy digested, food intake and the production of feces were determined as previously described (14). Cumulative food intake was measured every two days at 7-weeks-old fed ND or HFD diet and then calculated on a per day and per mouse basis. In week 7, 8, and 9, feces were collected twice after 48 h and dried to constant weight at 60°C, and energy content of dried feces (kJ/g) was analyzed for each individual using a bomb calorimeter (ZDHW-8LA, Yixin, China). Gross energy content of dried food was assessed by bomb calorimetry, and this value was subsequently used to determine individual energy intake (E_in_) per day. The amount of energy digested (E_dig_) per day was calculated from the difference between E_in_ and energy lost with feces (E_fec_).

### Small intestinal characterization

The entire small intestine (SI), from the gastric pylorus to the ileocecal valve, was dissected from anesthetized male WT and *Per1*^*−/−*^ mice (8-weeks-old). The SI length was measured and then opened longitudinally, washed in cold saline to clear the luminal contents, dried briefly on a paper towel, and weighed. The SI was divided 5 cm distal from the pyloric junction, and the jejunoileum was divided into equal proximal and distal fragments. Proximal fragments were fixed in 10% neutral-buffered formalin (NBF) for 24 h and sectioned (5 μm) for H&E staining. Villus height and crypt counts were determined using Image J software. Crypts in a 1-mm field were counted; 10 fields were analyzed per section. Villus height was measured from 10 well-oriented villi on each slide.

### Gastrointestinal motility

Total gastrointestinal transit time, gastric emptying, and small intestinal transit were measured as previously described (15). Briefly, total gastrointestinal transit time was estimated as the time required for a nonabsorbable dye (carmine red) to appear in stool after its gavage into the stomach. Gastric emptying and small bowel transit were evaluated following the gavage of rhodamine B dextran in methylcellulose. The gut was removed 15 min after gavage, and the percentage of dye remaining in the stomach as well as the geometric center of the rhodamine B dextran in the intestine was determined (16).

### Fat accumulation studies

For measuring temporal fat accumulation, 8-week-old male WT and *Per1*^−/−^ mice on ND were fed saline or olive oil (10 μl/g body weight) by gavage, respectively. For energy stress, 8-week-old male WT and *Per1*^−/−^ mice were deprived of food from ZT12–ZT24, and olive oil (10 μl/g body weight) was given by gavage next ZT0. For longtime high-fat stress, male WT and *Per1*^−/−^ mice fed a ND or HFD for 8 weeks. Fat mass was measured before and 5 h after oil gavage by NMR analysis with the Bruker Minispec. The difference of fat mas between before and after oil gavage was calculated as the fat mass gain. Fat mass gain indicates the oil induced fat accumulation in whole body. Blood and liver tissues were collected for analyzing lipids composition.

### Biochemical analyses

The animals were sacrificed, and blood was collected from a carotid artery. Tissues were collected, weighed, and immediately frozen in liquid nitrogen for further analysis. Serum was then separated by centrifugation at 3,000 *g* for 15 min and stored at −80°C until analysis. For hepatic TG and cholesterol measurements, liver lipids were extracted as described previously (17). Briefly, hepatic tissue was homogenized, 10% wt/vol, in isopropanol for 20 s. To extract triglycerides and cholesterol, the homogenate as kept at 4°C for 2 days and then centrifuged at 1,000 *g* for 10 min. Aliquots of the supernatants were analyzed for triglycerides and cholesterol. Concentrations of serum TG, TC, free fatty acid (FFA), HDL-C, LDL-C, hepatic TG, and TC were determined by an enzyme assay method using the commercialized Test Kits (Nanjing Jiancheng Bioengineering Institute, Nanjing, China). Serum insulin levels were determined by ELISA (Crystal Chem Inc).

### Histological analysis

Tissues were fixed in 10% NBF, embedded in paraffin, and sectioned (5 μm). H&E staining was performed using standard techniques. For H&E and oil red O (ORO) staining, liver tissues were collected from male WT and *Per1*^−/−^ mice fed a HFD for 8 weeks or olive oil (10 μl/g body weight) 2 h postgavage at ZT2 and ZT14.To visualize neutral lipids, tissues were stained in 60% of the oil red O stock solution (0.5 g oil red O in 100 ml isopropanol) for 30 min. Tissues were briefly washed in 60% isopropanol and then rinsed in distilled water for microscopic observation and photography. Adipocyte size was measured as described previously (18). Especially, intestine samples were flushed with 10% neutral buffered formalin to remove stool contents and fixed in at least 10 times their volume 10% NBF for 10 days at 4^o^C. Segments were then cryoprotected by overnight immersion in a 30% sucrose solution (wt/vol).The following day, the tissues were rinsed gently in distilled water, placed in Tissue-Tek O.C.T. embedding media (VWR, Bridgeport, NJ), and frozen at −80^o^C. Slides were stained with Oil-Red O for microscopic observation and photography. For assessment of gallbladder size, the area of gallbladder was analyzed using Image J analyzer software according to literature (19).

### Fecal fat analysis

Eight-week-old male WT and *Per1*^−/−^ mice were placed in an empty cage and then given by garage with saline or olive oil at ZT0 and ZT12. The collected stool between 3 and 5 h was dried to a constant weight, and the fats were extracted as described (20). Extracted fecal fats from 100 mg of dried feces were dissolved in 1 ml of chloroform, and 1 μl of pooled sample was spotted onto a Silica G TLC plate. The plate was developed and stained as described (21). Quantitative analysis of FFA was determined by an enzyme assay method using the commercialized Test Kits (Nanjing Jiancheng Bioengineering Institute, Nanjing, China).

### RNA extraction and quantitative real-time RT-PCR

Total RNA was extracted from the samples with Trizol (KarrotenScientific, Nanjing, China) according to the manufacturer’s instructions. The reverse transcription reaction was carried out using a reverse transcriptase kit according to the manufacturer’s protocol. Real-time PCR was performed, and the products were detected using the ABI 7300 Detection System with SYBR Green dye. The expression level of *Gapdh* was simultaneously quantified as an internal standard control. The sequences of all primers used for quantitative RT-PCR are shown in the supplemental Table S1.

### Western blot analysis

Proteins were extracted following the procedure described previously (22). The proteins were separated by SDS-PAGE on 8–12% polyacrylamide gels and subsequently electrically transferred to a PVDF membrane. After blocking with 5% (w/v) BSA in TBST at room temperature for 1 h, the membranes were then incubated with an appropriate specific primary antibody (anti-CYP7A1, Santa Cruz, Cat# sc-518007, 1:500; anti-PER1, Sigma-Aldrich, Cat# AB2201, 1:200; anti-CYP8B1, Santa Cruz, Cat# sc-101387,1:500; anti-CYP27A1, Santa Cruz, Cat# sc-390974,1:500; anti-HNF-4α, Santa Cruz, Cat# sc-101059,1:500; anti-PKA-C, Santa Cruz, Cat# sc-365615,1:500; anti-PKA-R, Santa Cruz, Cat# sc-271125,1:500; anti-β-actin, BioWorld, Cat# AP0060, 1:1000; anti-p-Ser, Santa Cruz, Cat# sc-81514) at 4°C overnight, followed by incubation with an HRP-conjugated secondary antibody (1:4000). Detection was performed using an enhanced chemiluminescence kit (Thermo Scientific, Hudson, NH).

### Co-immunoprecipitations

Co-immunoprecipitation was performed as described previously with slight modifications (23). Briefly in vivo, fresh livers were homogenized and lysed with a solution containing 10 mM Tris-HCl (pH 8.0), 420 mM NaCl, 1 mM EDTA, and 0.5% NP-40 with protease inhibitor cocktail (Boster Biological Technology Ltd). To prepare the immunoprecipitates, we incubated the lysates with specific antibody overnight at 4°C followed by incubation with Protein G-Sepharose 4B. The immunoprecipitates were washed five times with wash buffer containing 10 mM Tris-HCl (pH 8.0), 100 mM NaCl, 1 mM EDTA, 0.5% NP-40%, and 0.5% Triton X-100 and subsequently boiled in SDS-PAGE loading buffer. Antibodies to CYP7A1 (Santa Cruz, Cat# sc-518007, 1:10; Abcam, Cat# ab65596, 1:50), PER1 (Sigma-Aldrich, Cat# AB2201, 1:50), and CYP8B1 (Santa Cruz, Cat# sc-101387, 1:10) were performed for co-immunoprecipitation. The proteins were analyzed by Western blotting as described above.

### Bile acid analysis

Bile acids in liver, intestine (with content), and gallbladder were extracted with 95% ethanol overnight, 80% ethanol for 2 h, and methanol/chloroform (2:1) for 2 h at 50°C. Total bile acids were determined with a bile acid assay kit (Jiancheng Bioengineering Institute, Nanjing, China). Gallbladder bile acid compositions were analyzed using liquid chromatography/mass spectrometry as described previously (24).

### Microsomal enzyme activity assay

Microsomes are isolated from mice livers by differential ultracentrifugation (25)). The livers were removed quickly, perfused with 0.9% NaCl, and then homogenized with 4 volumes of 0.25 M sucrose containing 5 mM Tris-HC1 and 1 mM EDTA (pH 7.4). The homogenate was centrifuged at 600 g for 12 min and then at 9,000 *g* for 15 min in the cold. The supernatant layer was further centrifuged at 105,000 *g* for 60 min, and the resulting microsomal precipitate was washed once with 0.15 M KC1 containing 5 mM Tris-HC1 and 1 mM EDTA (pH 7.4) and suspended in 100 mM Tris-HC1 buffer (pH 7.4) containing 1 mM EDTA and 1 mM dithiothreitol.

CYP7A1 enzyme activity was determined as described (26). Liver microsomes are diluted in 1 ml of reaction buffer in a 50-ml, screw-capped Corex centrifuge tube. Ten microliters of 10 mM cholesterol in acetone is added. Samples are preincubated at 37°C for 5 min. The reaction is initiated with 100 μl of 10 mM NADPH for 15 min at 37°C with shaking. For control experiments, a sample is boiled for 3 min before adding NADPH. The reaction is terminated with the addition of 30 μl of 20% sodium cholate. Forty microliters of cholesterol oxidase (25 U/ml), suspended in 10 mM potassium phosphate, pH 7.4, 1 mM DTT, and 20% glycerol (v/v) is added and incubated for 10 min at 37°C. The reaction is stopped by adding 2 ml of ethanol and then extracted three times with 6 ml each of petroleum ether at 37°C. The extracted products in the top phase are evaporated to dryness in a reagent vial, tightly sealed, and stored under N_2_ in a desiccator at −20°C. Samples were analyzed using an HPLC (Waters 1525 System; Millipore, Bedford, MA) on a reversed-phase C18 column. The gradient elution started with 70% acetonitrile and 30% methano passing through the column at a flow rate of 0.8 ml/min. The variable-wavelength detector was set at 240 nm.

CYP8B1 enzyme activity was determined as described (27). Microsomes were incubated for 10 min at 37°C with 7α-hydroxy-4-cholesten-3-one (6 μg dissolved in 5 μl of isopropyl alcohol), NADPH (1 mM) and 100 mM potassium phosphate buffer (pH 7.4) containing 0.1 mM EDTA in a total volume of 0.3 ml. The incubation was stopped by addition of 5 ml of benzene. Formed 7α,12α-dihydroxy-4-cholesten-3-one was quantified by HPLC.

### Everted gut sac transport measurement

The mucosal-to-serosal transport of taurocholate was measured as described previously (28). After euthanization, a 7-cm segment of SI ileum was isolated after measuring from the beginning of the large bowel. The everted gut sac was prepared from this segment. The everted gut sac was filled with Kreb’s Ringer Buffer (KRB, pH 7.4), and taurocholate transport was determined (the mucosal-to-serosal direction) after immersing the everted gut sac into the mucosal fluid containing KRB supplemented with 25 mM taurocholate and a tracer ^3^H-taurocholate as well as ^14^C-inulin to normalize for paracellular leakage. The KRB bathing solution was continuously gassed with 95% O_2_/5% CO_2_. Radioactivity of ^3^H-taurocholate in the serosal fluid was measured after a 30 min interval.

### Emulsion preparation

Lipid emulsification capacity was measured as described previously with some modifications (29). The emulsions were prepared by mixing 40 μl olive oil into 100 μl buffer solution, and a fixed volume of fresh murine bile samples (obtained just after killing) was added to a final volume of 160 μl. The tubes were capped and shaken at 1,000 strokes/min for 20 min at 37°C. The samples were examined and photographed immediately after preparing the emulsion under bright field illumination on a photomicroscope. The procedure was duplicated by taking a second sample from the same emulsion.

### Statistical analysis

The single cosinor method was used for analysis of circadian rhythm, and the cosine function equation was as follows: Y (t) = M + Acos (xt + u). The rhythm characteristics estimated by this method included the mesor (middle value of the fitted cosine representing a rhythm-adjusted mean), the amplitude (half the difference between the minimum and maximum of the fitted cosine function), and the acrophase (time of peak value in the fitted cosine function). All data were shown as the means ± SD. The statistical analysis of the results was performed using GraphPad Prism 5 software (San Diego, CA). Statistical difference between groups was determined by Student’s *t* test, and comparisons among groups were performed using ANOVA.

## Results

### Daily fat absorption and accumulation is deregulated in *Per1*-knockout mice

**Fig. 1:**
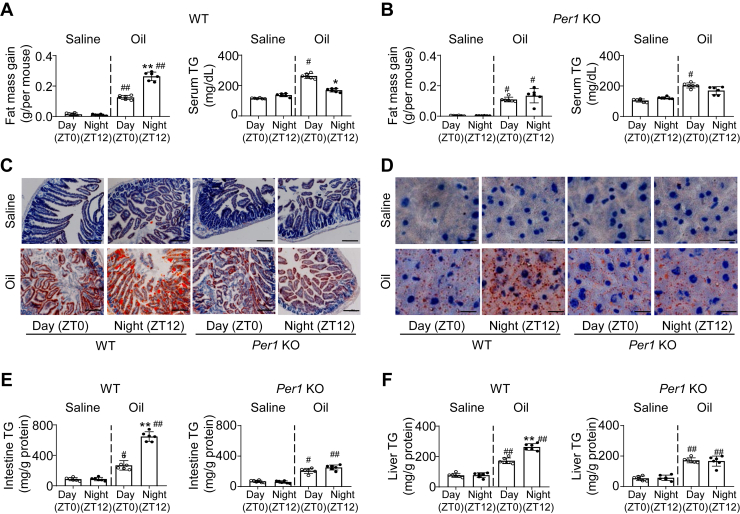
*Per1* deficiency deregulates temporal fat accumulation in mice. A, B: Male WT (A) and *Per 1* KO (B) mice (8-weeks-old) were gavaged with saline or olive oil (10 μl/g body weight) at ZT0 or ZT12, and fat mass gain (left) and serum TG (right) were measured after 5 h (n = 6). C, D: Representative ORO staining of proximal intestines and livers from male WT and *Per1* KO mice (8-weeks-old) following gavage with saline or olive oil after 2 h (n = 3 mice per group). Scale bar in intestinal section, 100 μm; scale bar in liver section, 25 μm. E, F: Male WT and *Per1* KO mice (8 weeks old) were gavaged with saline or olive oil (10 μl/g body weight) at ZT0 or ZT12, and intestinal (E) and hepatic (F) TG was measured after 5 h (n = 4–5 mice per group). Throughout, data are presented as the mean ±SD. ^#^*P* < 0.05, ^##^*P* < 0.01, oil versus saline group in WT and *Per1* KO mice; ∗*P* < 0.05, ∗∗*P* < 0.01, day versus night group. Analyses were performed using two-way ANOVA for (A, B, E, and F).

To examine the potential effects of feeding time on fat absorption and accumulation in mice, we initially searched for a difference in fat mass gains after gavage with olive oil at 7:00 during the daytime (ZT0) and at 19:00 during the nighttime (ZT12), respectively. Via an NMR analysis and TG measuring, we found that WT mice challenged with olive oil at ZT12 significantly increased both fat mass gain and serum triglycerides compared with mice at ZT0 (Fig. 1A). This day–night difference in fat accumulation could be seen in circadian *Per2*-deficient mice (supplemental Fig. S1A, B). Unexpectedly, this diurnal oscillating profile of fat accumulation was deregulated in mice deficient in *Per1*, a negative modulator like *Per2* in core clock system (30), displaying no obvious difference between day and night (Fig. 1B). To clarify this observation, we carried out histological analysis for fat absorption in the intestine and fat accumulation in the liver, respectively. Consistently, increased fat absorption and accumulation during the night (ZT12) was observed in the intestines and livers of WT mice with olive oil feeding (Fig. 1C–F), whereas *Per1*^*−/−*^ mice fed the same amounts of olive oil demonstrated no difference between day and night in fat absorption and accumulation (Fig. 1C–F). Therefore, these observations suggest a potential role of *Per1* in circadian-controlled daily fat absorption and accumulation.

### *Per1* deficiency weakens the capability of fat accumulation

**Fig. 2:**
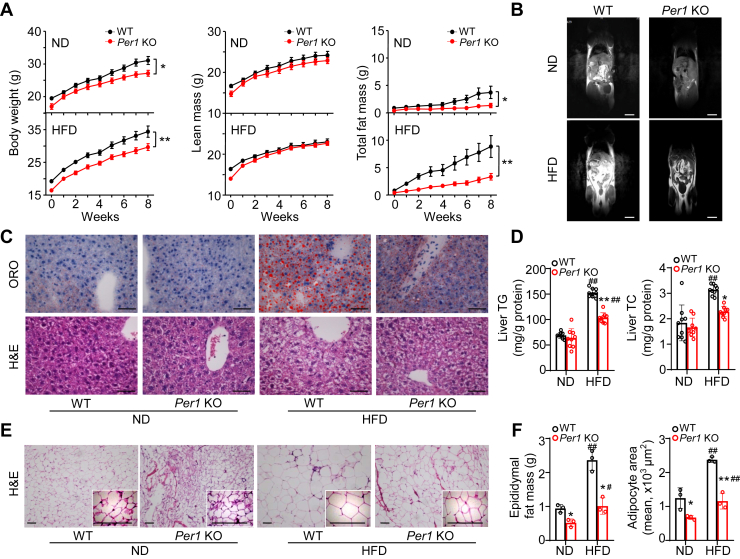
*Per1* deficiency decreases fat accumulation. A: Body weight (left), total fat mass (middle) and lean mass (right) were measured weekly in WT and *Per1* KO mice fed a normal diet (ND) or a high-fat diet (HFD) for 8 weeks (n = 9 mice per group). B: Magnetic resonance imaging of each genotype of mouse fed a ND or HFD for 8 weeks was performed with a preclinical MRI system on the T1 sequence. The bright parts represent visible body fat. n = 3 mice per group, representative plots are shown. Bar = 1 cm. C: Representative ORO imaging of livers from WT and *Per1* KO mice fed a ND or HFD for 8 weeks (n = 3 mice per group), and scale bar represents 100 μm. D: Hepatic TG and TC were measured in WT and *Per1* KO mice fed a ND or HFD for 8 weeks (n = 9 mice per group). E: H&E staining of epididymal fat from WT and *Per1* KO mice fed a ND or HFD for 8 weeks (n = 3 mice per group). Representative plots are shown, and the scale bar represents 50 μm. F: Epididymal fat mass weight and quantitative analysis of adipocyte area (n = 3 mice per group). Throughout, data are presented as the mean ± SD. ^#^*P* < 0.05, ^##^*P* < 0.01, HFD versus ND group in WT and *Per1* KO mice; ∗*P* < 0.05, ∗∗*P* < 0.01, *Per1* KO mice versus WT mice. Analyses were performed using linear mixed model for A; and two-way ANOVA for (D, F).

To determine the role of *Per1* in fat accumulation and obesity, we compared *Per1*-deficient mice to WT littermates fed a standard ND (10% kcal from fat) or a HFD (60% kcal from fat) for 8 weeks, respectively. Despite the diurnal rhythm in normal food intake was not obviously altered in the *Per1*^−/−^ animals (supplemental Fig. S2A–D), body weight and total fat mass were significantly less regardless of those fed on ND or HFD, compared with WT mice (Fig. 2A, left panel and right panel). No significant differences in lean mass (Fig. 2A, middle panel), serum TG, TC, and insulin level (supplemental Fig. S2E–I) were noted between both genotypes. An MRS assessment of total fat further confirmed a reduction of body fat composition in *Per1*^*−/−*^ mice (Fig. 2B). While fed on HFD, *Per1*-deficient mice demonstrated an obvious decrease in hepatic steatosis (Fig. 2C), liver TG, and TC accumulation (Fig. 2D). The epididymal fat of *Per1*^*−/−*^ mice showed a reduction in adipocyte size and a decrease in total fat mass (Fig. 2E, F). Together, these results indicate that *Per1* deficiency weakens the capability of fat accumulation.

### *Per1* deficiency impairs the capability of FA absorption in intestines

**Fig. 3:**
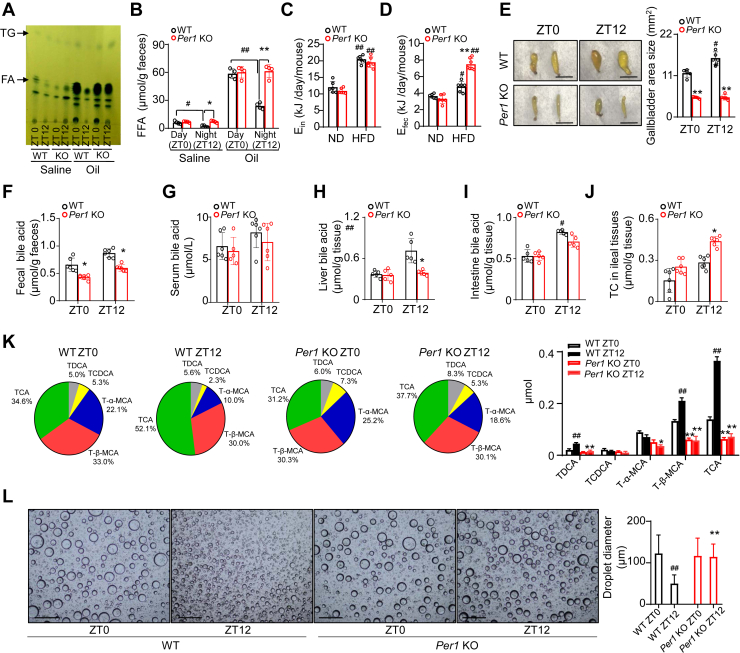
*Per1* deficiency leads to defective bile acid regulation. A: TLC analysis of FFAs in stools of male WT and *Per1* KO mice (8–10 weeks old). Fresh fecal samples were collected between 2–5 h after gavage with saline or olive oil at ZT0 and ZT12. Pooled samples (n = 4/time point/genotype) were used for each time point. B: Quantitative analysis of FFAs in stools between genotypes (n = 4 per group). ^#^*P* < 0.05, ^##^*P* < 0.01, day versus night group in WT mice; ∗*P* < 0.05, ∗∗*P* < 0.01, *Per1* KO versus WT mice. C, D: Daily energy intake (E_in_) (C) and daily energy loss (E_fec_) (D) from male WT and *Per 1* KO mice fed a ND or HFD for 3 weeks (n =6 per group). ^#^*P* < 0.05, ^##^*P* < 0.01, HFD versus ND group in WT and *Per1* KO mice, respectively; ∗∗*P* < 0.01, *Per1* KO mice versus WT mice. E: Representative photos of gallbladder from WT and *Per1* KO mice at ZT0 or ZT12. Bar = 5 mm. Gallbladder area sizes were measured using Image J in both genotypes at ZT0 or ZT12 (n = 5). F, I: Bile acid levels in feces (F), serum (G), livers (H), and intestine (I) were measured in male WT and *Per1* KO mice at ZT0 or ZT12 (n = 5). J: Bile acid reabsorption in ileal fragment of small intestine was measured using a probe bile acid ^14^C-taurocholic acid. n = 5 per group. ^#^*P* < 0.05, ^##^*P* < 0.01, day versus night group in WT mice; ∗*P* < 0.05, ∗∗*P* < 0.01, *Per1* KO versus WT mice. K: Gallbladder bile acid composition analysis. Pie charts of the mean percentage of detected bile acids in WT and *Per1* KO mice. Gallbladder concentration of individual bile acid species were shown in WT and *Per1* KO mice. n = 4. L: Representative microscope images of droplets in an emulsion. The emulsifying capacity of the bile acids from WT and *Per1* KO mice at ZT0 or ZT12 was determined by in vivo experiments (scale bars, 500 μm). Average droplet size under each condition were measured by Image J. ^#^*P* < 0.05, ^##^*P* < 0.01, day versus night group in WT mice; ∗*P* < 0.05, ∗∗*P* < 0.01, *Per1* KO versus WT mice. Throughout, data are presented as the mean ± SD. Analyses were performed using two-tailed Student's *t* test for B; two-way ANOVA for (C, D, F, G, H, I, J, K, and L).

To determine whether *Per1* deficiency affects diurnal dietary fat absorption, we compared the amount of fat and FA in the feces of WT and *Per1*^*−/−*^ mice between day and night, respectively. A thin layer chromatography analysis revealed that the feces from mice gavaged with saline or olive oil had the same appearance and contained about a much little proportion of TG regardless of the genotypes and feeding time (Fig. 3A, top). While the results for TG were less clear, .a day–night variation pattern with higher levels at ZT0 signifying reduced lipolysis during the day could be seen (Fig. 3A, top). However, in *Per1*^*−/−*^ mice, the differences in TG levels between day and night were much reduced (Fig. 3A, top). When WT mice were given olive oil at ZT0 during the daytime, FFA comprised a much greater proportion of feces compared to that at ZT12 during nighttime. In *Per1*^*−/−*^ mice, FFA predominated the same great proportion in the feces obtained from day and night (Fig. 3A, middle). A precise measuring for fecal FFA confirmed a day–night difference of FFA absorption present in WT mice, and this difference was abolished when mice lost *Per1* (Fig. 3B), reflecting that *Per1* could play an irreplaceable role in diurnal FFA absorption in the intestine endothelium (Fig. 3A, B). Next, we found that the energy intake (E_in_) of *Per1*^*−/−*^ and WT mice did not vary much under ND and HFD (Fig. 3C). However, energy content of feces (E_fec_) was consistently higher in *Per1*^*−/−*^ mice compared with WT mice fed on HFD (Fig. 3D), confirming *Per1*^*−/−*^ mice impaired ability of intestinal fat absorption. We then detected that SI weight and length, villus height and crypt number, total gastrointestinal transit time, and gastric emptying did not differ between genotypes (supplemental Fig. S3A–H), indicating *Per1* deficiency had no influence on intestine morphology and motility. Next, we examined whether circadian *Per1* directly impaired transcriptions of some key target genes involved in FFA absorption. Quantitative RT-PCR analysis revealed *Per1* deficiency failed to decline the expression of the genes related to FA and TG synthesis, FFA transport, TG re-synthesis, and assembly of chylomicrons in SIs (supplemental Fig. S4). Although a potential effect of these genes on diurnal absorption of FA could not be completely excluded, this factor alone does not appear to play a major part in the lower FA absorption observed in *Per1*^*−/−*^ mice. Together, these results indicate *Per1* deficiency impairs FA absorption in intestines.

### *Per1* deficiency attenuates circadian synthesis of bile acid

Within the SI, the ingested TGs are emulsified by BAs and hydrolyzed into monoglycerides and FFAs by specific esterases (31).Intraluminal BA concentrations have a profound effect on FA digestion and absorption. To determine the effect of BAs on day–night difference of FA absorption, the total BA pool size was examined at special time points. The size of gallbladders did have a robust diurnal pattern with a bigger size at ZT12 and smaller at ZT0 in WT mice (Fig. 3E). A significantly smaller gallbladder in *Per1*^*−/−*^ mice at ZT0 and ZT12 could be seen in comparison with WT mice (Fig. 3E). Consistent with this reduction in gallbladder size, fecal and liver BA levels were reduced in *Per1*^*−/−*^ mice (Fig. 3F, H). Confusingly, there were no significant differences between WT and *Per1*^*−/−*^ mice in the intestine and serum (Fig. 3G, I). Most BAs are reabsorbed in the ileum and are transported back to the liver via portal blood circulation (32). Then we assessed reabsorption of BAs in WT and *Per1*^−/−^ mice by using the ileal everted gut sac model. We observed that *Per1* deficiency displays an exaggerated increase in the mucosal-to-serosal transport of taurocholate (Fig. 3J). We next assayed the BA levels in these mice on HFD. Serum and liver BA levels of WT mice on HFD were significantly elevated over those on ND (supplemental Fig. S5A, B). *Per1*^*−/−*^ mice had significantly lower liver BA levels than WT animals when kept on HFD (supplemental Fig. S5B). Intestinal BA levels of *Per1*^*−/−*^ mice were essentially indistinguishable from that of control littermates (supplemental Fig. S5C). Interestingly, while the composition of the gallbladder BAs varied between ZT0 and ZT12 in WT mice (Fig. 3K), *Per1*^*−/−*^ mice have markedly reduced taurocholic acid compared with WT mice (Fig. 3K). BAs composition determines the emulsifying capacity, which emulsifies fat and fat-soluble nutrients and is essential for their absorption. We also tested the emulsifying capacity of BAs from these mice. Specifically, the samples treated with BAs from WT mice at ZT12 had significantly more small-sized lipid droplets and significantly fewer large-sized lipid droplets (Fig. 3L). Comparing to WT mice, the samples supplemented with BAs from *Per1*^*−/−*^ mice had significantly more large lipid droplets and fewer small lipid droplets at ZT12 (Fig. 3L). Collectively, these findings identify *Per1* as an essential regulator for rhythmic variations of BA pools and compositions.

### Cholic acid treatment partly restores fat absorption and accumulation in *Per1*-knockout mice

**Fig. 4:**
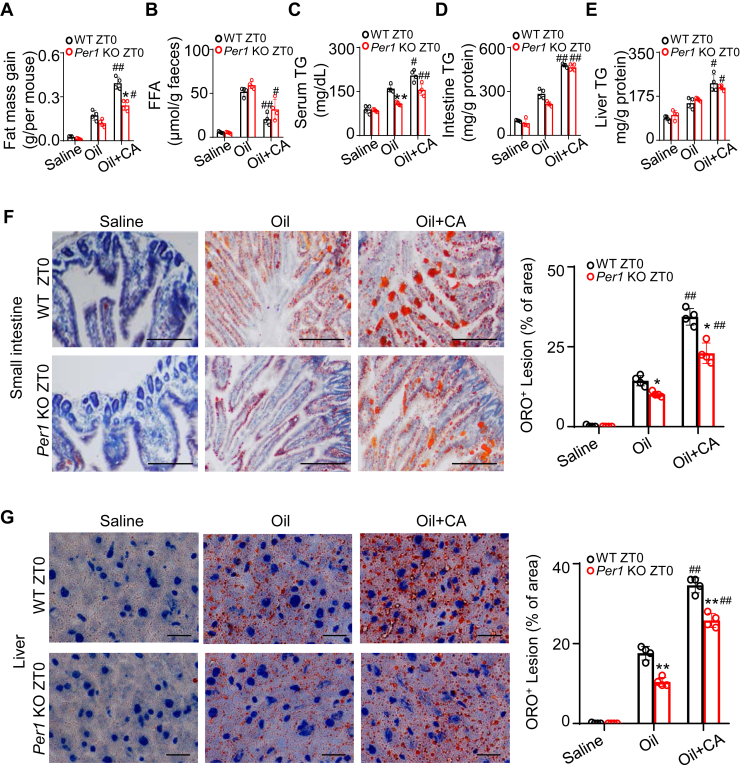
CA administration partly rescues the deficiency of fat absorption in *Per1* KO mice. A: Male WT and *Per* 1 KO mice were gavaged olive oil (10 μl/g body weight) with or without 150 μg/g cholic acid (given simultaneously with olive oil) at ZT0. Saline group mice were gavaged with saline as a control. Fat mass gain was measured after 5 h. n = 4 per group. B: Quantitative analysis of FFAs in stools between genotypes 5 h after gavage. n = 4 per group. C, E: Serum TG (C), intestine TG (D) and liver TG (E) in mice 5 h after gavage. (n = 5 WT and 4 *Per1* KO mice). F to G: Micrographs showing ORO staining of small intestine (F) and liver (G) in the indicated groups (red = Oil Red O, blue = hematoxylin, scale bar in intestinal section, 100 μm; scale bar in liver section, 25 μm). Percentage of ORO staining was determined using Image J (n = 4). Throughout, data are presented as the mean ± SD. Analyses were performed using two-tailed two-way ANOVA. ∗*P* < 0.05, ∗∗*P* < 0.01, *Per1* KO versus WT mice. ^#^*P* < 0.05, ^##^*P* < 0.01, Oil+CA versus Oil group.

In humans and mice, one of the most abundant bile acids is cholic acid (CA) (33). To further test whether the difference of day–night in fat absorption and accumulation is through *Per1* regulating the BA homeostasis, we administered CA to mice of both genotypes at ZT0. At this time point, the mRNA expression of the *Per1* gene in the liver of WT mice is at its minimum, and WT mice have lower levels of bile acids. We found that CA plus oil-treated WT mice showed more fat mass gain than oil-treated WT mice at ZT0 (Fig. 4A). Similarly, CA treatment rescued lipid absorption in *Per1*^*−/−*^ mice (Fig. 4A). *Per1*^*−/−*^ mice were restored to a near-normal level after CA treatment (Fig. 4A). Next, we assessed lipid absorption by measuring fecal lipid content. Compared with oil-treated mice, the amount of FFA in the feces was reduced in CA-treated group (Fig. 4B). CA also reduced residual FFA in feces of *Per1*^*−/−*^ mice and were comparable to those in oil-treated WT mice (Fig. 4B). CA-treated mice challenged with olive oil significantly increased serum, intestine, and liver fat accumulation in both genotypes at ZT0 (Fig. 4C–E). Staining with ORO further confirmed a massive lipid deposition in intestines and livers of mice gavaged with oil, which were largely diminished in the intestines and livers of *Per1* knockout mice (Fig. 4F, G). ORO staining revealed that lipid droplet accumulation in intestines and livers of CA-treated *Per1*^−/−^ mice was significantly increased (Fig. 4F, G). The above results suggested that the administration of CA partially restores the absorption and accumulation of fat in *Per1*-knockout mice.

Moreover, chenodeoxycholic acid treatment failed to increase lipid absorption in both genotypes at ZT0 (supplemental Fig. S6A) and could not restore lipid absorption of *Per1*^*−/−*^ mice at ZT12 (supplemental Fig. S6B). TG levels in serum, liver, and intestine of *Per1*^*−/−*^ mice had no significant changes after chenodeoxycholic acid treatment (supplemental Fig. S6C–E). These results further validate our predictions that circadian variation of BA pools and compositions is responsible for day–night difference of fat absorption and accumulation.

### The regulatory mechanism of *Per1*/PKA-mediated phosphorylation on CYP7A1 and CYP8B1 activities

**Fig. 5:**
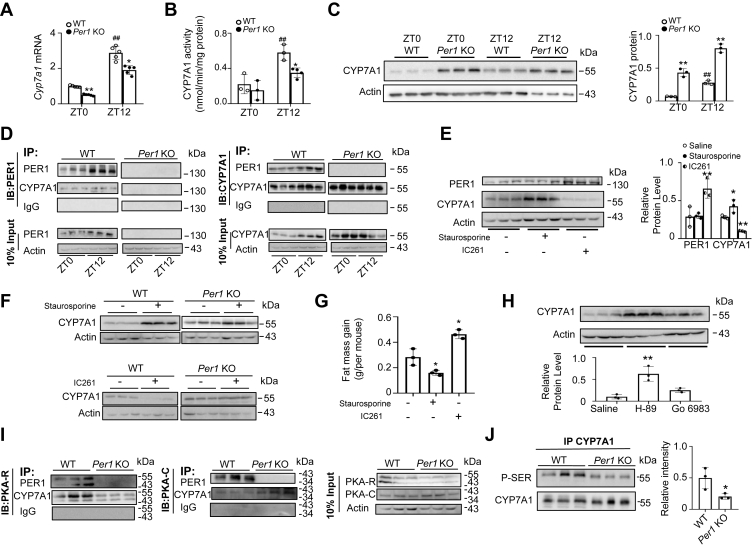
PER1 regulates CYP7A1 activity via PKA. A: Quantitative PCR quantification of *Cyp7a1* mRNA, normalized to *Gapdh* mRNA (n = 5 per group). B: Quantitative analysis of CYP7A1 activity in WT and *Per* 1 KO mice (n = 3 per group). C: Western blotting analysis of CYP7A1 in liver tissue obtained from WT and *Per* 1 KO mice (n = 3). β-actin was used as internal control. ^#^*P* < 0.05, ^##^*P* < 0.01, day versus night group in WT mice; ∗*P* < 0.05, ∗∗*P* < 0.01, *Per1* KO versus WT mice. D: Representative co-immunoprecipitation results of PER1 and CYP7A1 interaction from extracts of WT and *Per1*^*−/−*^ mouse liver at ZT0 or ZT12. β-actin as an input control. n = 3 per group. IB, immunoblot; IP, immunoprecipitate. E: Western blot of CYP7A1 and PER1 from WT mice treated with staurosporine (2 μg/g body weight) or IC261 (30 μg/g body weight) via intraperitoneal injection, respectively. n = 3 per group. F: Western blot of CYP7A1 from *Per1*^*−/−*^ mice treated with staurosporine or IC261, respectively. n = 3 per group. G: The mice within each group were subjected to either staurosporine or IC261 and underwent the olive oil gavage (10 μg/g body weight) at ZT6. Fat mass gain were measured after 5 h. n = 4 per group. ∗*P* < 0.05, ∗∗*P* < 0.01 versus control mice. H: Protein levels of CYP7A1 in H-89 (20 μg/g body weight) or Go 6893 (2.3 μg/g body weight) treated mice were shown by Western blot. All drugs were given by intraperitoneal injection. β-actin was used as loading control. The correspondent quantification was shown. n = 3 per group, ∗*P* < 0.05, ∗∗*P* < 0.01, versus control mice. I: Co-IP of PKA-C and PKA-R with PER1 and CYP7A1 in WT and *Per1* KO mice. Reciprocal Co-IP was conducted using PER1 or CYP7A1 antibody for IP, respectively. n = 3 per group. IB, immunoblot; IP, immunoprecipitate. J: IP done using CYP7A1 antibody, and the levels of phospho-serine (p-Ser) were determined using phospho-specific antibodies as indicated. ∗*P* < 0.05, ∗∗*P* < 0.01, *Per1* KO versus WT mice. Throughout, data are presented as the mean ± SD. Analyses were performed using two-tailed two-way ANOVA.

To determine the cause of the changes of BA pool and composition, we analyzed the expression of the rate-limiting enzyme in the BA synthesis by quantifying the expression of *Cyp7a1*, which catalyzes the initial step in the neutral synthetic pathway of BAs from cholesterol. Hepatic mRNA level for *Cyp7a1* displayed diurnal variation in WT mice, and the *Per1* deletion reduced *Cyp7a1* mRNA (Fig. 5A), and the activity was significantly reduced in *Per1*^−/−^ mice (Fig. 5B). However, while we examined CYP7A1 protein levels in WT and *Per1*^−/−^ mice, the result was unexpected as *Per1* deficiency significantly increased CYP7A1 protein levels (Fig. 5C), suggesting that *Per1*-dependent changes in CYP7A1 protein appears independent of transcription.

We then performed Co-IP assays to investigate the binding of PER1–CYP7A1 proteins. As shown in Fig. 5D, PER1 is directly bound to CYP7A1, and the binding efficiency exhibited obvious diurnal changes in the liver of WT mice but not in *Per1*^−/−^ mice. We next retrieved site-specific information for CYP7A1 from PhosphoSitePlus (http://www.phosphosite.org), and the data revealed multiple phosphorylation sites in CYP7A1 proteins. To further test whether PER1 regulates CYP7A1 activity through a phosphorylation-dependent mechanism, we examined the effects of several kinase inhibitors. Staurosporine is a promiscuous kinase inhibitor targeting all kinase families (34) and thus would be expected to affect CYP7A1. Western blotting showed that staurosporine led to a robust increase in CYP7A1 protein levels, while PER1 levels remained unchanged (Fig. 5E). PER1 contains a nuclear localization signal adjacent to a binding site for casein kinase 1 epsilon (35). With casein kinase 1 inhibitor IC261 treatment, CYP7A1 protein level gradually decreased with increasing PER1 (Fig. 5E). For further validation, we treated *Per1*^*−/−*^ mice with staurosporine and IC261. CYP7A1 protein levels of WT mice significantly increased following treatment with staurosporine, consistent with the level of CYP7A1 in *Per1*^−/−^ mice (Fig. 5F). We found that IC261 was inactive in the absence of *Per1*, showing IC261 works by targeting PER1 protein (Fig. 5F). We subsequently examined the effects of kinase inhibitors on lipid absorption. Whereas the broad-spectrum kinase inhibitor staurosporine reduced fat mass gain after oil treatment, IC261 increased fat mass gain (Fig. 5G). These results suggest that *Per1* regulates phosphorylation of CYP7A1 by kinases. To further define the target kinase, we measured CYP7A1 protein levels in mice treated with PKA inhibitor H-89 or PKC inhibitor Go 6983. As shown in Fig. 5H, H-89 strongly induced the CYP7A1 protein, whereas Go 6983 did so very weakly. To determine whether PKA was the direct binding target, we used Co-IP assays and showed that PKA associates with PER1 and CYP7A1 (Fig. 5I). We confirmed the strong PER1–PKA interaction and PKA was able to bind both PER1 and CYP7A1 in WT mice (Fig. 5I). The results from *Per1*^−/−^ mice showed that CYP7A1 protein could bind PKA even without PER1 (Fig. 5I).

The phosphorylation of CYP7A1 by IP using anti-CYP7A1 antibody followed by immunoblotting for antiphosphorylation serine (p-Ser) antibody was reduced in *Per1*^−/−^ mice (Fig. 5J). PER1 appears to affect BA synthesis signaling by regulating the catalytic activity of the bound CYP7A1 protein, by regulating interactions between the bound CYP7A1 protein and PKA protein or by controlling the localization of the bound CYP7A1 protein. Next, we observed the similar effects of PER1 on CYP8B1, another key protein for CA (supplemental Fig. S7). These results suggest that *Per1* regulates CYP7A1 and CYP8B1activity via a PKA-dependent pathway.

### Increased fat absorption under fasting and high-fat stress depends on *Per1* action

**Fig. 6:**
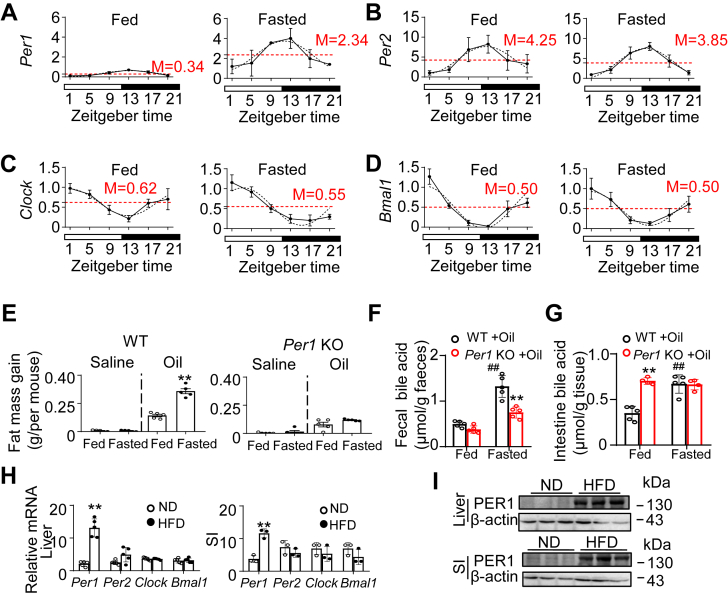
Fasting and high-fat diet increases fat absorption and accumulation depending on *Per1* action. A–D: Mice were fasted up to 12 h, starting at ZT0 and sacrificed at ZT1, ZT5, ZT9, ZT13, ZT17, and ZT21. Hepatic mRNA levels of *Per1* (A), *Per2* (B), *Clock* (C), and *Bmal1* (D) were measured by real-time PCR (n = 5 per group). Solid lines indicate experimental response curves; dashed lines, fitted model curves. Red dashed line with the number represents mesor. E: Fat mass gain was measured in WT and *Per1*^*−/−*^ mice (8-weeks-old) undergoing fasting stress from ZT12 to ZT24, following olive intake next ZT0 (n = 5). ∗*P* < 0.05, ∗∗*P* < 0.01 Fasted compared with Fed group. F, G: Bile acid levels in feces (F) and intestine (G) were measured in male WT and *Per1* KO mice (8-weeks-old) undergoing fasting stress from ZT12 to ZT24, following olive intake next ZT0. n = 5 per group, ^#^*P* < 0.05, ^##^*P* < 0.01, Fasted compared with Fed group; ∗*P* < 0.05, ∗∗*P* < 0.01, *Per1* KO versus WT mice. H: The expression profiles of clock genes in the livers and small intestines of ND mice compared to HFD mice. Quantitative RT-PCR was performed for analyzing mRNA level in ND (white) and HFD (black) mice. n = 5 per group, ∗*P* < 0.05, ∗∗*P* < 0.01 compared with lean group. I: Western blot analysis of PER1 protein level in livers and small intestines of ND mice compared to HFD mice (n = 3). β-actin was used as internal control.

To investigate the potential role of *Per1* on fat accumulation in energy stress, we examined the fat accumulation behavior of mice fed ad libitum with that of mice subjected to metabolic stress. Fasting stress was generated by food deprivation for 12 h from ZT12 to ZT24. As shown in Fig. 6A, the results confirmed that fasting stress acutely increased the expression of *Per1* in livers (Fig. 6A). While *Per1* mRNA levels dramatically elevated, the mRNA rhythmicities of other clock genes were maintained in mice livers (Fig. 6A–D). These findings revealed that fasting-induced liver stress resulted in changes in the expression of circadian clock genes with a specific elevation of *Per1* mRNA. To further identify the ability of *Per1* to regulate accumulation of lipid, we measured the changes in fat mass gain between the no stress and fasting stress treatment. Twelve-hour fasting stress increased lipid absorption in WT mice (Fig. 6E). Thus, fasting stress was not sufficient to restore lipid absorption in *Per1*^*−/−*^ mice, indicating that the presence of *Per1* is necessary for lipid absorption under stress. At this time, the BA levels in feces and intestines were tested. The BA levels in feces and intestines were greater in fasted WT mice than in fed WT mice (Fig. 6F, G). By contrast, *Per1* deficiency did not result in such changes (Fig. 6F, G). The levels of BA in feces were lower in *Per1*^*−/−*^ mice compared to WT mice (Fig. 6F). It was unexpected that the levels of BA in intestines were not reduced in *Per1*^*−/−*^ mice, although this may reflect a substantial change in BA composition. Moreover, in HFD mice, *Per1* mRNA and protein levels, but not other clock genes, were significantly elevated in the intestines and livers compared to ND mice at ZT0 (Fig. 6H, I). Thus, our findings support the results of the studies in *Per1*-deficient mice and suggest a role for the PER1 gene in the regulation of fat absorption and accumulation in humans.

## Discussion

The main conclusion from our results is that circadian *Per1* is a major driven factor in regulating diurnal intestinal fat absorption and accumulation. Rhythmic *Per1* expression is thought to be part of a negative feedback loop generating circadian oscillations of cellular functions in nucleus (30). Our data demonstrated that circadian PER1 directly bound PKA to associate with CYP7A1 and CYP8B1 proteins and modulated their modification and activity. *Per1*-driven fluctuations of CYP7A1 and CYP8B1 activity caused daily variations of BA pool size and composition involved in lipid emulsification, influencing FFA uptake and resulting in temporal fat absorption in intestines and fat accumulation in livers.

In humans, the evening eaters are 2-fold more likely to be obese than morning eaters while eating the same number of calories (9). The persons assigned to high caloric intake during breakfast lose significantly more weight than those assigned to high caloric intake during dinner (36). Another study shows that the timing of the main meal is predictive of the weight loss conducted in obese and overweight individuals, and the effect is independent of the total 24-h caloric intake (11). These studies clearly show a day–night difference in the capacity of fat accumulation and energy storage. It is known that the intestines harbor functional peripheral circadian clocks similar to liver, and *Per* (*Per1* and *Per2*) expressions display robust rhythmicity with low peak during the day and high peak during the night, respectively (37). Our current data show the profile of fat absorption in intestine tightly correlates with the phase of the *Per1* and *Per2* expression rhythms, and this diurnal rhythmic intestinal absorption of fat is abolished in *Per1*-deficient mice but not in *Per2*-deficient mice, suggesting that *Per1* is a specific regulator in intestinal absorption of fat.

The FFAs are emulsified with the help of BAs to form mixed micelles which are then taken up by the enterocytes lining the villi of the SI. Early studies in humans have demonstrated that BA concentrations show a diurnal rhythm and peak after food ingestion in the systemic circulation (38, 39). The BA concentrations in serum, liver, intestine, and gallbladder exhibited diurnal rhythms in mice (40). *Cyp7a1* is the rate-limiting enzyme in converting cholesterol to Bas (41). Within the classic pathway, CYP7A1 is the rate-limiting enzyme while CYP8B1 regulates the synthesis of CA and thus regulates the BA pool composition (42). Studies in the rats have shown that the activities of the hepatic *Cyp7a1* govern BAs synthesis and have diurnal variation, reaching a peak at midnight and thereafter gradually decreasing to a minimal activity at noon (43, 44, 45, 46, 47, 48, 49). The *Cyp8b1* mRNA levels also showed circadian change with the degree much smaller than those of *Cyp7a1* (50). Although the diurnal changes of *Cyp7a1* and *Cyp8b1* seem to be regulated at the transcriptional level (47, 51) and the key enzymes involved in bile acid synthesis are regulated by the nuclear receptor FXR (33), our present study indicated the posttranslational modification of *Cyp7a1* and *Cyp8b1* may be more important compared with transcriptional regulation. Our study points to the presence of a complex network in the regulation of CYP7A1 and CYP8B1 function by phosphorylation. PKA-mediated CYP7A1/8B1 phosphorylation is a major switch for the activity of BA synthesis by endogenous circadian PER1 interaction.

Some defined CYPs are phosphorylated in an isozyme-selective manner by PKA (52, 53). The PKA recognition motif Arg–Arg-X–Ser is present in several members of the CYP2 family such as CYP2B1/2B2 and CYP2E1 (52). Phosphorylation of CYPs is a very fast process, and the function of phosphorylated protein is significantly changed (53). It is shown that a substantial portion but not the entire pool of CYP2B1 molecules is phosphorylated, and the phosphorylated portion is catalytically fully inactive (52). Our findings suggest CYP7A1/8B1 phosphorylation could stimulate its enzyme activities and increase the instability of protein structure.

It is known that several proteins have diurnal variations in their phosphorylation. Of more than 20,000 phosphosites, 25% significantly oscillate in the mouse liver, including novel sites on core clock proteins (54). The tyrosine phosphorylation of HNRNPQ was rhythmic and showed a reciprocal profile to mPER1 (55). Ultradian rhythms in liver gene expression and AKT phosphorylation emerge in the absence of environmental rhythms and *Per1,2* genes (56). Reduced levels of phosphorylated CREB have been observed in *Per1* knock-out mice (57). The binding of PER1 protein to RSK in mouse hippocampal neurons is necessary for RSK translocation to the cell nucleus and learning-induced CREB phosphorylation, suggesting that clock proteins may regulate protein function through mechanisms beyond transcriptional control (58). Our previous study identifies that PER1 enhanced GPx activity through PER1/GPX1 interaction in cytoplasm, consequently improving the oxidative phosphorylation efficiency of mitochondria (59). PER1 can bind to phosphorylated checkpoint kinase 2, and the phosphorylation of the checkpoint kinase 2 Thr-68 site is blocked following ionizing radiation in the *Per1* siRNA-transfected HCT116 cells (60). In present study, we revealed a novel molecular mechanism underlying that PKA-mediated CYP7A1/8B1 phosphorylation is a major switch for BAs synthesis by endogenous circadian PER1 interaction, causing the rhythmic variations of BA pools, fat absorption, and accumulation.

Changing feeding schedules seems to disturb diurnal fat absorption and accumulation. Dietary fat absorption is highly efficient in WT mice (61). From our observations, fat absorption could be affected by feeding time. A previous study have showed that while administering corn oil–containing [^3^H] trioleate to mice, a significant amount of radioactivity is present in feces (62). Mice fed with an HFD only during the day weigh significantly more than those fed only during the night (10). Notably, in this experiment, before being fed with an HFD during the day, mice were deprived of food and fasted for 12 h from ZT12 to ZT24. This change of feeding schedule causes an energy stress and significantly disturbs normal circadian gene expression, with a special elevation of absolute levels of *Per1* over 24-h including daytime in multiple peripheral organs (63, 64). Indeed, from our data, while WT mice were fasted overnight following olive oil intake in the morning, a significant increase in fat mass gain was observed. This stress-related fat accumulation cannot be detected in fasting *Per1*^*−/−*^ mice. Our results also revealed obese mice and people trend to absorb and accumulate more fat due to high fat–induced *Per1* overexpression. A growing body of literatures suggests that social stress, broadly defined as an individual’s negative response to environmental pressure, is one contributor to the development and maintenance of obesity both in adults and youth (65, 66). The circadian system must continuously adapt to and synchronize our physiology with the environment, and *Per1* promoter has been proved to act as a sensor for multiple signaling molecules including PKA, PKC, and cAMP responsive element binding protein, thereby integrating different physiological parameters for rapid adaptation to changing environmental conditions (67). Thus, circadian *Per1* is a potential candidate of a key regulator in stress response and the relevant obesity risk.

## Data availability

All data are contained within the manuscript.

## Supplemental data

This article contains supplemental data.

## Conflict of interest

The authors declare that they have no conflicts of interest with the contents of this article.
