# Supplementary material for: Circadian PER1 controls daily fat absorption with the regulation of PER1-PKA on phosphorylation of bile acid synthetase
Source: J Lipid Res. 2023 May 18;64(6):100390. doi: 10.1016/j.jlr.2023.100390 (PMC10276160; doi:10.1016/j.jlr.2023.100390)
Supplement: Supplemental Figures S1–S8 and Table S1 [file mmc1.docx]

**Supplementary data**

**Circadian PER1 Controls Daily Fat Absorption with the Regulation of PER1-PKA on Phosphorylation of Bile Acid Synthetase**

Wenhao Ge^1^†, Qi Sun^2^†, Yunxia Yang^1^, Zhao Ding^1^, Junhao Liu^1^, Jianfa Zhang^1^*

From the ^1^Center for Molecular Metabolism, Nanjing University of Science & Technology, Nanjing, 210094, China; ^2^Key Laboratory of Cardiovascular and Cerebrovascular Diseases, Bengbu Medical College, Bengbu, 233030, China.

†These authors contributed equally to this study.

*Correspondence to Jianfa Zhang, [jfzhang@mail.njust.edu.cn](mailto:jfzhang@mail.njust.edu.cn), Center for Molecular Metabolism, Nanjing University of Science & Technology, 200 Xiaolingwei Street, Nanjing, China, Tel: 86-25-84318533.

**The** **supplementary data includes:**

1. Table **S**1
2. Figure **S**1-8

**Table. S1 qPCR primers-Related to RT-qPCR**

| *Real-time PCR Primers* | | |
| --- | --- | --- |
| GENE | Forward Primer | Reverse Primer |
| *Mouse* | | |
| *Acc1* oligos: | GCCATTGGTATTGGGGCTTAC | CCCGACCAAGGACTTTGTTG |
| *Acsl3* oligos: | TGTCTTTCTCATGGATGCCGA | CAGCACGGATGTGTCTCCTT |
| *Acsl5* oligos: | CAGTGGAACTACAGGTGACCC | TACCCTGGACAAGCCTCTCA |
| *ApoAIV* oligos: | GCACAACAAGCTGGTGCCC | CAGGTGCTCCTGCAACTTCTG |
| *ApoA1* oligos: | GATGCGGTGGAACACTTTCT | ACTGTCCTCGACTTCCGAGA |
| *ApoB* oligos: | GATCAGGCTTTGCCGCAATA | CATCAGGAGAGGCCAATCC |
| *Bmal1* oligos: | ACATAGGACACCTCGCAGAA | AACCATCGACTTCGTAGCGT |
| *Clock* oligos: | CGGCGAGAACTTGGCATT | AGGAGTTGGGCTGTGATCA |
| *Cyp7a1* oligos: | GCCTCTGAAGAAGTGAATGG | TAAAAGTCAAAGGGTCTGGG |
| *Cyp8b1* oligos: | CTGGCTTCCTGAGCTTATTC | CCCAGTAGGGAGTAGACAAA |
| *Dgat1* oligos: | TGCTCTTTTTCACCCAGCTT | TTGAAGGGCTTCATGGAGTT |
| *Fat/cd36* oligos: | GCCAAGCTATTGCGACATGATTA | ATCCGAACACAGCGTAGATAGAC |
| *Fasn* oligos: | GGACATGGTCACAGACGATGAC | GACGACTGACAGCACTTGGA |
| *Fabp1* oligos: | CATCCAGAAAGGGAAGGACA | CTTCCCTTCATGCACGATTT |
| *Gapdh* oligos: | CATCCACTGGTGCTGCCAAGGCTGT | ACAACCTGGTCCTCAGTGTAGCCCA |
| *Hsl* oligos: | TCACGCTACATAAAGGCTGCT | CCACCCGTAAAGAGGGAACT |
| *Ptl* oligos: | CCTGATGACGCTGATTTTGT | TATGCTTTGCTGGGGTTTTC |
| *Mgat2* oligos: | TGGGAGCGCAGGTTACAGA | CAGGTGGCATACAGGACAGA |
| *Mttp* oligos: | CCTCTTGGCAGTGCTTTTTC | ATTTTGTAGCCCACGCTGTC |
| *Per1* oligos: | TCCTCAACCGCTTCAGAGATC | CGGGAACGCTTTGCTTTAGA |
| *Per2* oligos: | GTGAAGCAGGTGAAGGCTAAT | AAGCTTGTAAGGGGTGGTGTAG |
| *Srebp1c* oligos: | GGAGCCATGGATTGCACATT | GGCCCGGGAAGTCACTGT |
| *Scd1* oligos: | CCCCTGCGGATCTTCCTTAT | AGGGTCGGCGTGTGTTTCT |

**Fig. S1** **Intestinal absorption of fat in *Per2* deficiency mice.** (**A** and **B**) Male *Per2^-/-^* mice (8 weeks old) were given saline (**A**) or olive oil (**B**) by gavage once at ZT0 or ZT12, five hours later, fat mass gain was measured. Data were expressed as mean ± s.e.m; n=5 per group. **p*<0.05 compared with the day group. Male mice for this experiment were maintained on standard chow.

**Fig. S2 Feeding behavior and serum analysis in WT and *Per1^-/-^* Mice.** (**A**-**E**) Food and water intake were measured every two day for male WT and *Per1^-/-^* mice fed a ND or HFD for 8 weeks (n=9). Mean food intake (**A**) and mean water intake (**B**) were expressed as grams per day and body weight. Food intake (**C**) and water intake (**D**) during the daytime and nighttime per body weight were measured for two weeks in ND (n=9). No significant difference in food intake was observed between WT and *Per1^-/-^* mice. (**E**-**I**) Serum TG (**E**), TC (**F**), HDL-C (**G**), LDL-C (**H**) and insulin (**I**), were analyzed in male WT and *Per1^-/-^* mice fed a ND or HFD for 8 weeks (n=9). No significant difference was observed between WT and *Per1^-/-^* mice. All data were expressed as mean ± s.e.m.

**Fig. S3 *Per1* deficiency does not affect small intestine morphology and motility.** (**A**-**H**) The average weight (**A**) and length (**B**) of the small intestine was similar between WT and *Per1^-/-^* mice (n=6, 3 months old). Total GI transit time (GIT) (**C**), gastric emptying (**D**) and small intestinal transit time (geometric center is a proxy) (**E**) was not affected between WT and *Per1^-/-^* mice (n=6, 6-8 weeks old). Proximal intestinal samples were H&E stained (**F**; scale bar, 150μm), representative plots are shown; villus height (**G**; n=6) and crypt numbers (**H**; n= 6) have no significant differences between genotypes. All Data were expressed as mean ± s.e.m and male mice for all experiments were maintained on standard chow.

**Fig. S4 *Per1* deficiency does not affect genes related to FFA absorption and lipoprotein assembly.** The expression profiles of the genes related to FFA absorption and lipoprotein assembly in the small intestine of WT and *Per1^-/-^* mice (6-8 weeks old). Diurnal mRNA level of in the intestines of WT (black) and *Per1^-/-^* (red) mice were determined by quantitative real-time RT-PCR (n=4/time point/genotype). The white and black bar represents day and night, respectively. No significant differences in these genes expressions were observed between genotypes. All data were expressed as mean ± s.e.m and male mice for all experiments were maintained on standard chow.

**Fig. S5 Changes of bile acid pool change in HFD-fed *Per1* deficiency** **mice.** (**A**-**C**) Graph showing bile acid levels in serum (**A**), livers (**B**), and intestine (**C**) in the WT and *Per1* KO mice fed on ND, HFD (n=5 mice/group). **p*<0.05, ***p*<0.01, *Per1* KO mice versus WT mice. All data were expressed as mean ± s.e.m and male mice for all experiments were maintained on standard chow.

**Fig. S6** **CDCA administration did not affect lipid absorption in *Per1* deficiency mice.** (**A**-**B**) Male WT and *Per* 1 KO mice were gavaged olive oil (10 µl/g body weight) with or without 150 µg/g CDCA (given simultaneously with olive oil) at ZT0 (**A**) and ZT12 (**B**). Saline group mice were gavaged with saline as a control. n=4 per group. (**C**-**E**) Serum TG (**C**), intestine TG (**D**), and liver TG (**E**) in *Per* 1 KO mice gavaged olive oil (10 µl/g body weight) with or without 150 µg/g cholic acid (n=5 WT and 4 *Per1* KO mice) at ZT12. ^#^*p*<0.05, ^##^*p*<0.01, Oil versus Saline group; **p*<0.05. No significant difference was observed between Oil+CDCA and Oil group. Throughout, data are presented as the mean ± s.e.m. Analyses were performed using two-tailed two-way ANOVA.


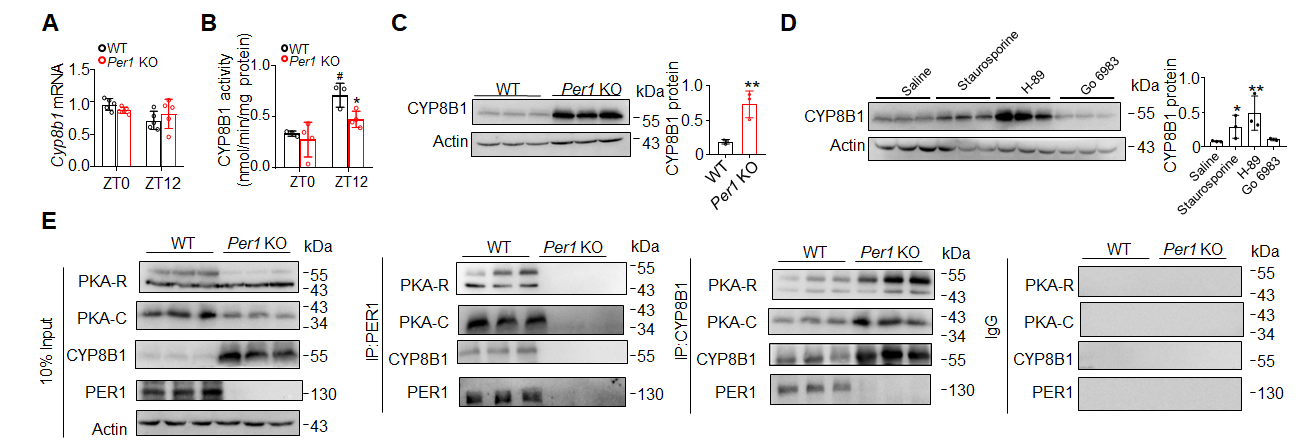


**Fig. S7 PER1 regulates CYP8B1 activity via PKA.** (**A**) Quantitative PCR quantification of *Cyp8b1* mRNA, normalized to *Gapdh* mRNA (n=5 per group). (**B**) Quantitative analysis of CYP8B1 activity in WT and *Per* 1 KO mice (n=3 per group). (**C**) Western blotting analysis of CYP8B1 in liver tissue obtained from WT and *Per* 1 KO mice at ZT12 (n=3). ^#^*p*<0.05, ^##^*p*<0.01, day versus night group in WT mice; **p*<0.05, ***p*<0.01, *Per1* KO versus WT mice. (**D**) Protein levels of CYP8B1 in staurosporine (2 µg/g body weight), H-89 (20 µg/g body weight) or Go 6893 (2.3 µg/g body weight) treated mice were shown by western blot. All drugs were given by intraperitoneal injection. β-actin was used as loading control. The correspondent quantification was shown. n=3 per group, **p*<0.05, ***p*<0.01, versus control mice. (**E**) Co-IP of PKA-C and PKA-R with PER1 and CYP8B1 in WT and *Per1* KO mice. Reciprocal Co-IP was conducted using PER1 or CYP8B1 antibody for IP, respectively. n=3 per group. Throughout, data are presented as the mean ± SD. Analyses were performed using two-tailed two-way ANOVA.


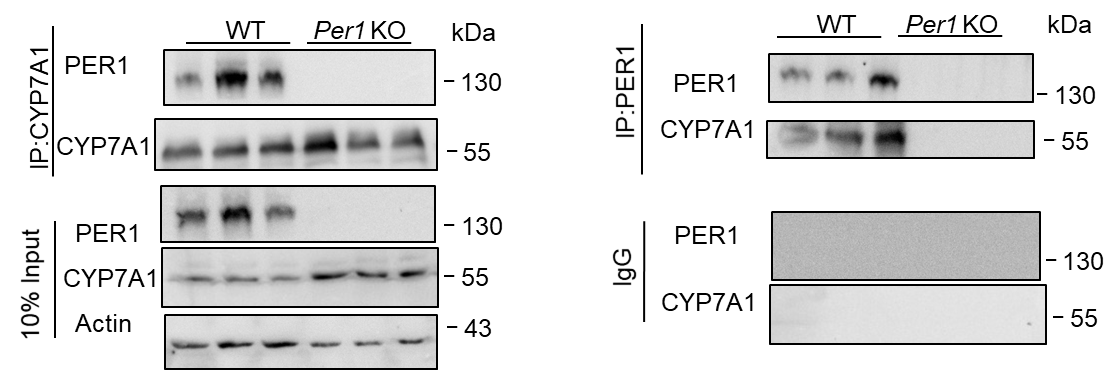


**Fig. S8 PER1 binds to CYP7A1.** Representative co-immunoprecipitation results of PER1 and CYP7A1 interaction from extracts of WT and *Per1*^−/−^ mouse liver at ZT12. β-actin as an input control. n=3 per group. IP, immunoprecipitate.
